# Expression Profiling and Cell Type Classification Analysis in Periodontitis Reveal Dysregulation of Multiple lncRNAs in Plasma Cells

**DOI:** 10.3389/fgene.2020.00382

**Published:** 2020-04-28

**Authors:** Donglei Wu, Peng Zhou, Fengdi Cao, Zhengshen Lin, Defeng Liang, Xincai Zhou

**Affiliations:** ^1^Department of Stomatology, Shenzhen Baoan Women’s and Children’s Hospital, Jinan University, Shenzhen, China; ^2^Department of Stomatology, Guangdong Women and Children Hospital, Guangzhou, China; ^3^Department of Stomatology, The First Affiliated Hospital of Jinan University, Guangzhou, China

**Keywords:** periodontitis, differential immune-related genes, immune infiltration, long non-coding rna, gene set enrichment analysis

## Abstract

**Objective:**

Periodontitis is a chronic inflammatory disease with a downregulated immune response. The mechanisms of the immune response, especially regarding immune-related long non-coding RNAs (lncRNAs), in periodontitis remain unclear. This study aimed to analyze the immune cell landscapes and immune-related transcriptome expression in periodontitis.

**Materials and Methods:**

The periodontitis-related microarray data set GSE16134 was downloaded from the Gene Expression Omnibus database. Then, the proportions of the infiltrated immune cell subpopulations were evaluated by Cell-type Identification By Estimating Relative Subsets Of RNA Transcripts (CIBERSORT). Differentially expressed immune-related genes (DEMGs) and lncRNAs were analyzed by the “limma” package in R software. Co-expression of DEMGs and lncRNAs in immune cell subpopulations was evaluated. Gene set enrichment analysis (GSEA) was performed to identify alterations in immune function through potential pathways.

**Results:**

Increased numbers of plasma cells were observed in periodontitis-affected tissues versus those of healthy tissues, while T cells were downregulated. A total of 51 DEMGs were identified, and 12 immune-related signaling pathways were enriched by GSEA, most of which were related to the stimulation and function of B cells and T cells. Only 3 differentially upregulated lncRNAs (FAM30A, GUSBP11, and LINC00525) were screened for the regulation of the immune response. Besides, the level of lncRNAs (FAM30A, GUSBP11, and LINC00525) expression were positively correlated with the fraction of plasma cells in periodontitis.

**Conclusion:**

The discovery of differentially expressed immune-related transcriptomes in periodontitis lesions helps to explain the regulation of the immune mechanism in the development of periodontitis.

## Introduction

Periodontitis, a chronic inflammatory oral disease, is initiated by the oral microbiota in the periodontal pocket and stimulates host immune responses. The hallmark of periodontitis is continuously supporting bone loss with inflammatory infiltration and even tooth loss. As a common multifactorial disease, periodontitis is synergistically affected by heredity, environment (microbiota) and lifestyles (including smoking, diet and stress), and contributes to the disruption of immune homeostasis (Schaefer, 2018). However, different intensities of immune responses between individuals were found despite similar environments and lifestyles. Hence, genetic alterations encoding immune-related molecules in periodontitis have a profound impact on the specificity and sensitivity of the host to periodontal microorganisms.

Emerging evidence has suggested that long non-coding RNAs (lncRNAs) are linked to alterations in immune-related genes and cell subpopulations, including regulating T cells through FOXP3 (Zemmour et al., 2017), controlling dendritic cell differentiation through STAT3 binding to lncDC (Wang et al., 2014) and activating the differentiation of CD4 + and CD8 + T cells (de Lima et al., 2019). However, the role of lncRNAs in the immune response in periodontitis remains unknown.

We aimed to calculate the immune cell landscapes in gingival tissues with periodontitis by Cell-type Identification By Estimating Relative Subsets Of RNA Transcripts (CIBERSORT) software. Then, we performed transcriptome analysis in gingival tissues from public databases to identify immune-related mRNAs and lncRNAs. Moreover, we deduced that immune-related mRNAs might change the immunological pathways. The combination analysis between immune cell subpopulations and immune-related transcriptome analysis would provide new insights into the immunomodulatory mechanism of periodontitis.

## Materials and Methods

### Data Collection and Eligibility Criteria

Raw data regarding human gingival tissues or peripheral blood samples with periodontitis gene expression were retrieved from publicly available gene expression datasets (GSEs)from the Gene Expression Omnibus (GEO). Only five related gene datasets (GSE16134, GSE79705, GSE27993, GSE23586, and GSE6751) were obtained. GSE16134, GSE79705, GSE27993 and GSE23586 included human gingival tissue samples, while GSE6751 included peripheral blood samples. To ensure statistical power, a sample size of less than 50 or subjects with systemic diseases (such as diabetes) were excluded.

Due to the relatively small samples in GSE79705 (*n* = 8), GSE27993 (*n* = 10) and GSE23586 (*n* = 6) versus GSE16134 (*n* = 310) and inadequate types of peripheral blood mononuclear cells (PBMCs) in GSE6751, only GSE16134 was included in this study (Supplementary Figure S1). Subsequently, background adjustment and normalization in GSE16134 were conducted using R package (affy).

### Immune Cell Infiltration With CIBERSORT

CIBERSORT^1^ was applied to characterize the immune cell composition of gingival tissues based on a validated leukocyte gene signature matrix containing 547 genes and 22 human immune cell subpopulations (Supplementary Table S2; Newman et al., 2015). These immune cell subpopulations included naive B cells, memory B cells, plasma cells, seven types of T cells, monocytes, resting NK cells, activated NK cells, three types of macrophages, resting dendritic cells, activated dendritic cells, resting mast cells, activated mast cells, eosinophils and neutrophils. Normalized gene expression profiles of GSE16134 were input in CIBERSORT for analysis based on a deconvolution algorithm with 100 permutations. To control the accuracy of the deconvolution algorithm, data with a CIBERSORT *P*< 0.05 were screened for the next analysis. Both sensitivity and specificity in CIBERSORT signature based on 3061 human transcritomes were over 95% with area under curve (AUC) > 0.98, which denotes a robust evidence for predicting the immune cells composition.

### Immune-Related Gene Definition

The immune-related gene list was downloaded from the Immunology Database and Analysis Portal (Immport)^2^, which contains 1233 immune-related genes (Bhattacharya et al., 2014; Supplementary Table S1). After taking the intersection between differentially expressed genes in GSE16134 and immune-related genes with R, the differentially expressed immune-related genes (DEMGs) were filtered when | log (FC)| > 1 and FDR < 0.05 were statistically significant.

### Gene Set Enrichment Analysis

To further investigate the differentially expressed immune-related genes in the alteration of immunological pathways, gene set enrichment analysis (GSEA) (Subramanian et al., 2005) was performed to investigate the potential immune-related biological pathways in gingival tissues with periodontitis. The immune-related gene set was downloaded from the Immport database, which included 29 immune categories with different molecular functions. Immune-related pathway enrichment was identified with a false discovery rate (FDR) < 0.25.

### Identification of Immune-Related lncRNAs

Probes expression of lncRNAs in the GSE16134 profile were annotated based on the GENCODE database^3^. Differentially expressed lncRNAs were selected with the R package (limma). Pearson correlation analysis was conducted between immune cell types and DEMGs, between differentially expressed lncRNAs and DEMGs and between immune cell types and lncRNAs. Absolute values of the Pearson correlation coefficient (*r*) *>* 0.6 and a *P <* 0.05 were considered medium strong correlations. The visual co-expression network was conducted with Cytoscape software 2.8 (Kohl et al., 2011). Subsequently, the relationship between immune cell types and immune-related lncRNAs was calculated by Pearson analysis with the absolute value of *r >* 0.6 and *P <* 0.05.

### Quantitative Real-Time-PCR Validation

To verify the expression of immune-related lncRNAs (FAM30A, GUSBP11, and LINC00525) in periodontitis lesions. Twenty-seven gingival tissues with periodontitis lesions from patients diagnosed with periodontitis and 23 healthy gingival tissues from patients with tooth extraction for orthodontics treatment were analyzed. Informed consent was obtained from all participating individuals; the study was approved by institutional boards at Shenzhen Baoan Women’s and Children’s Hospital.

Total RNAs from the above samples were extracted by the TRIzol reagent (Invitrogen) according to the manufacturer’s guidance. By using the PrimeScript^TM^ RT Reagent Kit with gDNA Eraser (Takara Bio Inc., Shiga, Japan), extracted RNAs were reverse transcribed into complementary DNA (cDNA) in accordance with the manufacturer’s procedure. Real-time PCR was conducted by SYBR Premix Ex Taq^TM^ II (Takara) and the Applied Biosystems 7500 Real-time PCR System (Applied Biosystems, Inc., Carlsbad, CA, United States). Through the 2-ΔΔCt method, the relative expressions of target genes were calculated. Internal references were GAPDH and U6.

All specific primers were shown as follows: FAM30A forward primer 5′-TTGAATAGAGTAGTTCCTTGCGCTG-3′; FAM30A reverse primer 5′-GGCTACTTCACCCAGCTGTCTAG-3′; GUS BP11 forward primer 5′-TCCCCTGTCCCGAAGGATTAC-3′; GUSBP11 reverse primer 5′-TAAGGGACTAACGGCTTCG CT-3′; CARMN forward primer 5′-ATGCACACTTCTCGGC TAAGAGTC-3′; CARMN reverse primer 5′-CTACAATGCCAC AAGTGATTCCAGC-3′; LINC00525 forward primer 5′-TCTTTATCATCGATGCCAA-3′; LINC00525 reverse primer 5′-TCTACTAAGCTCGTTTCAA-3′.

### Statistical Analysis

Comparisons between two groups were determined by using a two-sided Wilcoxon test. Concordance among the immune cell type relative fraction was determined by the Pearson correlation coefficient to measure the degree of linear fit, and the root mean squared error (RMSE) was used to evaluate the estimation bias. Heatmaps were conducted by using the R software “pheatmap” package. Statistical analyses were conducted with the R package. Results with a *P* < 0.05 were considered statistically significant (Newman et al., 2015; Ge et al., 2019).

## Results

### Patient Characteristics

Only one gene expression dataset (GSE16134) was screened out. A total of 310 samples (67 healthy and 243 diseased) were included after CIBERSORT filtration. The included subjects with gingival tissue bleeding upon probing, a probing pocket depth ≥ 4 mm and clinical attachment loss ≥ 3 mm were identified as having periodontitis.

### Plasma Cells Infiltration in Periodontitis Tissues Versus Unaffected Tissues

**FIGURE 1:**
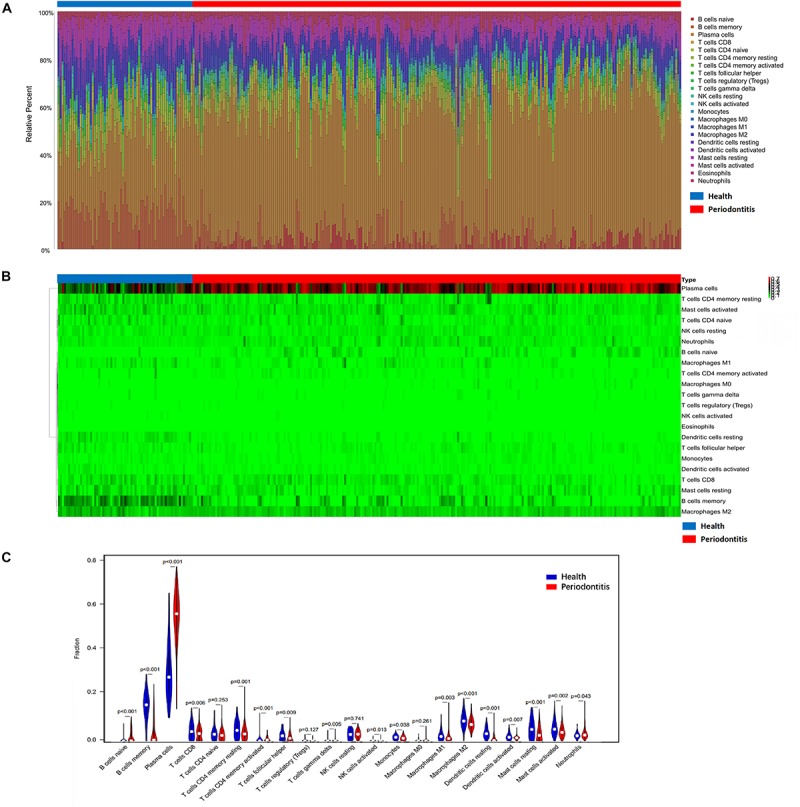
Composition of infiltrated immune cell subpopulations in gingival tissues with or without periodontitis. **(A)** Fraction of 21 infiltrated immune cell subpopulations determined by CIBERSORT. **(B)** Heatmap of the gene expression of immune cell subpopulations. **(C)** Comparison of infiltrated immune cell subpopulations in gingival tissues with or without periodontitis. A high proportion of plasma cells infiltrated in periodontitis individuals. Plasma cells increased in gingival tissues with periodontitis lesion versus those of healthy tissues.

Periodontitis tissues were infiltrated with affluent plasma cells in gingival tissues. Increased numbers of B cells, especially plasma cells, naïve B cells, and neutrophils, were observed in periodontitis-affected tissues, while the other immune cell types (including T cell subtypes, NK cells, monocytes, macrophages, dendritic cells, and mast cells) were depleted in number. Over 50% of plasma cells and 20% B lymphocytes infiltrated periodontitis lesions versus those of healthy gingival tissues. Rather, a low proportion of T cells was observed in “diseased” gingival tissues. Both the composition of M1 macrophages and M2 macrophages showed a decline in their number. No eosinophilic granulocytes were detected from the CIBERSORT results (Figure 1).

### Correlation Analysis Between Immune Cell Subpopulations

**FIGURE 2:**
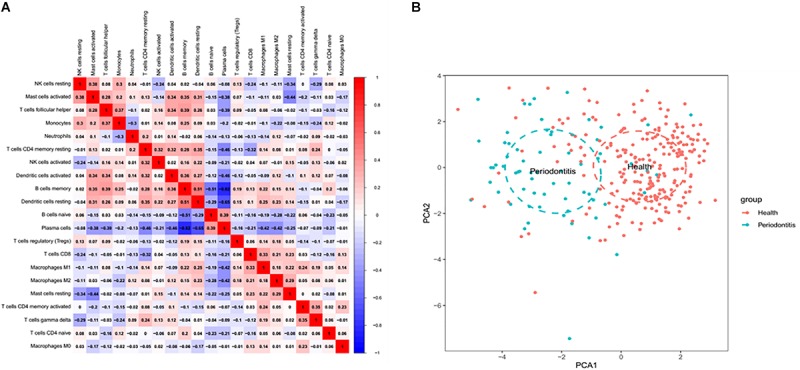
Coherence analysis of immune cell subgroups in gingival tissues with periodontitis. **(A)** Correlation among immune cell subpopulations. A negative correlation was observed between plasma cells and CD4 memory T cells and between resting DC cells and memory B cells. **(B)** Principal component analysis between healthy and periodontitis tissues based on infiltrated immune cell types.

There was no significant correlation between immune cell subpopulations (Figure 2). Positive correlation between memory B cells and follicular helper T cells, activated mast cells and resting NK cells, activated mast cells and memory B cells, and activated mast cells and activated dendritic cells is presented in Figure 2A. However, the number of plasma cells was negatively correlated with memory B cells (*r* = −0.82), activated dendritic cells (*r* = −0.46), resting dendritic cells (*r* = −0.65), memory resting CD4 + T cells (*r* = −0.46), M1 macrophages (*r* = −0.42) and M2 macrophages (*r* = −0.42). Principal component analysis (PCA) based on the infiltrated immune cell types revealed a distinguishing outcome between periodontitis and healthy tissues in Figure 2B.

### Identification of Differentially Expressed Immune-Related Genes

**FIGURE 3:**
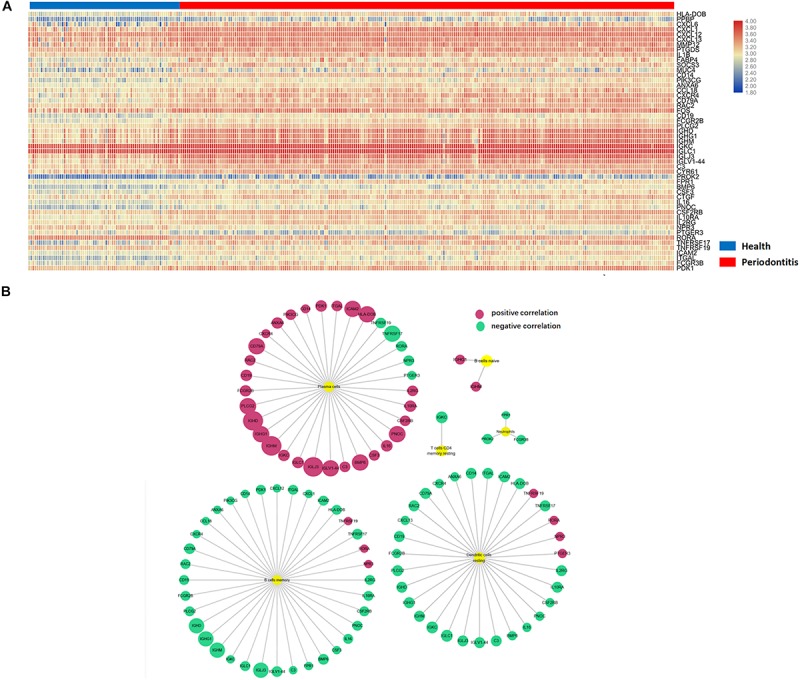
Differentially expressed immune-related genes in immune cells. **(A)** Differentially expressed immune-related genes in the heatmap. **(B)** Six immune cell subpopulations significantly correlated with DEMGs, especially for plasma cells, memory B cells and resting DCs.

Differentially expressed genes (DEGs) were identified according to the abovementioned selection criteria. There were 56 up- and 197 downregulated DEGs in gingival tissues affected with periodontitis versus those in unaffected tissues. After intersection with immune-related genes, 51 differentially expressed immune-related genes (DEMGs) were identified. Nearly all DEMGs were upregulated, except NPR3, PTGER3, RORA, and ICAM2. A heatmap of gene clustering analysis of the two groups (health and periodontitis) was shown in Figure 3A. In addition, the PCA analysis indicated that there was a significant disparity in DEMGs between health and periodontitis group (Supplementary Figure S2).

### Differentially Expressed Immune-Related Genes in Immune Cells

To further investigate the relationship between DEMGs and immune cell subpopulations, a Pearson analysis was conducted to reveal the strength of the correlation. There were six immune cell types (plasma cells, memory B cells, resting DCs, neutrophils, naive B cells and memory resting CD4 + T cells) that were significantly correlated with DEMGs. Gene expression of IGHD, IGLJ3, IGHM, IGLV1-44, IGHG1, BMP6, and PNOC were positively related to the number of plasma cells. In addition, the gene expression of RORA, NPR3, and TNFRSF19 were negatively correlated with the number of plasma cells and were inversely expressed in memory B cells and resting DCs. Interestingly, a negative correlation was found between TNFRSF17 and three immune cells (plasma cells, memory B cells, resting DCs) (Figure 3B).

### Immune-Related Pathway Enrichment in Periodontitis

**FIGURE 4:**
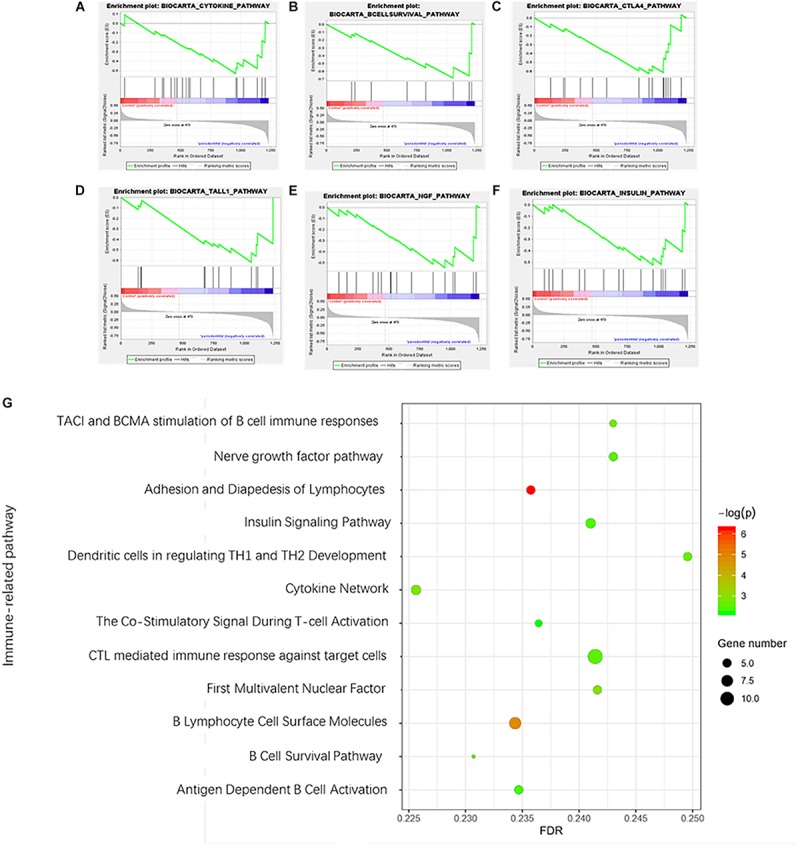
Gene set enrichment analysis of immune-related pathways in periodontitis. **(A)** Cytokine network pathway; **(B)** B cell survival pathway; **(C)** Costimulatory signal during T-cell activation; **(D)** TACI and BCMA stimulation of the B cell immune response pathway; **(E)** Nerve growth factor pathway; **(F)** Insulin signaling pathway. **(G)** Bubble diagram of enriched immune-related pathways.

A total of 12 immune-related pathways were enriched by GSEA (Figure 4). All pathways were negatively correlated with the presence of periodontitis. The immune-related pathways of the cytokines network played an important role in the development of periodontitis. Most immune-related pathways were concentrated on the stimulation and function of B cells and T cells. The immune-related pathway of TACI and BCMA stimulation of B cell immune responses confirmed the vital role of autoimmune disorders in periodontitis. Interestingly, a negative correlation between the nerve growth factor (NGF) pathway and insulin signaling pathway was identified in periodontitis, which suggested a relationship between systemic diseases and periodontitis.

### Immune-Related lncRNAs in Gingival Tissues With Periodontitis

**FIGURE 5:**
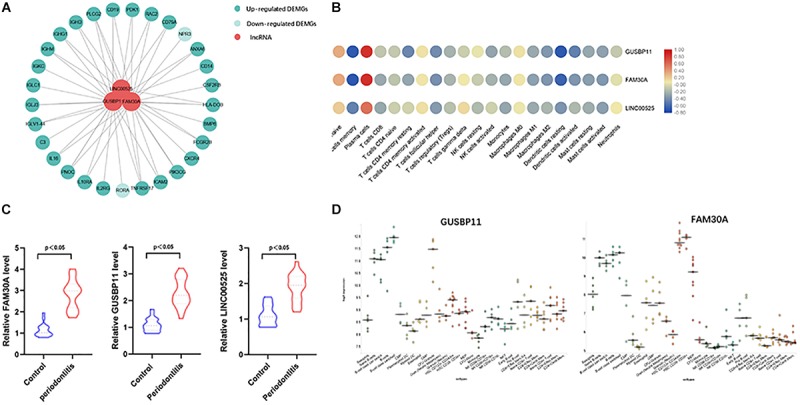
Immune-related lncRNAs identified in immune cell types. **(A)** Network analysis between differentially expressed lncRNAs and immune-related genes in gingival tissues with or without periodontitis. The connection between immune cell types and immune-related lncRNAs means the correlation coefficient with | R| > 0.6 and *P* < 0.05. Three immune-related lncRNAs (FAM30A, GUSBP11, and LINC00525) were identified. **(B)** Correlation analysis between immune-related lncRNAs and immune cell subpopulations. **(C)** RT-PCR validation of immune-related lncRNAs (FAM30A, GUSBP11, and LINC00525); **(D)** FAM30A and GUSBP11 expression profiles in normal human immune cells. lncRNAs (FAM30A and GUSBP11) were highly expressed in B cells and their terminally differentiated offspring.

A total of 1645 lncRNAs were expressed in GPL570 platform. Only 4 differentially upregulated lncRNAs (FAM30A, GUSBP11, CARMN, and LINC00525) were screened (Table 1). Clearly, FAM30A was the most significantly upregulated lncRNA with a logFC of 2.3. Three immune-related lncRNAs (FAM30A, GUSBP11, and LINC00525) were identified after co-expression with DEMGs. A total of 29 network notes and 52 interactive connections among 3 lncRNAs and 29 DEMGs were presented in the visual network expression profile, including LAM30A connecting with 23 DEMGs, GUSBP11 connecting with 22 DEMGs, and LINC00525 connecting with 7 DEMGs. The PCR results, consistent with the microarray chip data, demonstrated that all the differentially expressed lncRNAs (FAM30A, GUSBP11, and LINC00525) were upregulated in periodontitis compared with those in controls (Figure 5 and Supplementary Table S1).

**TABLE 1:** Differential expression of lncRNAs between periodontitis and healthy gingival tissues.

**lncRNA**	**logFC**	**P.Value**	**adj.P.Val**	**B**
FAM30A	2.332728	2.30E-31	1.89E-28	60.48085
GUSBP11	1.230011	2.06E-34	3.39E-31	67.38843
CARMN	1.229579	1.48E-28	8.13E-26	54.11162
LINC00525	1.002184	8.63E-12	4.05E-10	16.22549

Furthermore, lncRNAs (FAM30A, GUSBP11, and LINC00525) expression were highly consistent with the number of plasma cells (Table 2). However, the lncRNAs (FAM30A and GUSBP11)were negatively correlated with the number of resting DCs. Consistently, FAM30A and GUSBP11 were highly expressed in B cells and their terminally differentiated offspring in normal human immune cells according to the Bloodspot dataset (Bagger et al., 2018; Figure 5D).

**TABLE 2:** Correlation between lncRNAs and immune cell types.

**Immune cell type**	**lncRNA**	***P***	***R***
Memory B cells	GUSBP11	1.33E-45	−0.69257437
Memory B cells	FAM30A	2.25E-48	−0.707609487
Plasma cells	GUSBP11	1.09E-88	0.852302683
Plasma cells	FAM30A	7.31E-92	0.859699542
Resting Dendritic cells	GUSBP11	5.28E-47	−0.700292083
Resting Dendritic cells	FAM30A	3.26E-37	−0.640632168

## Discussion

### Lymphocyte Subpopulations Play a Vital Role in the Progress Periodontitis

The current study firstly described the 21 immune cell subpopulations, particularly plasma cells, which infiltrated gingival tissues with periodontitis lesions by CIBERSORT. Lymphocyte subpopulations play a vital role in the acceleration of periodontitis. Previous research demonstrated that plasma cells accelerated periodontal bone loss in periodontitis. Consistent with previous studies (Seymour and Greenspan, 1979; Berglundh et al., 2007), B lymphocytes dominated in periodontitis tissue lesions. Given that activated B cells promote osteoclastogenesis, their upregulation contributes to the development of periodontitis by producing numerous osteoclastogenic cytokines, including tumor necrosis factor alpha (TNF-α), interleukin 6 (IL-6), macrophage inflammatory protein-1 alpha (MIP-1α), and Monocyte chemoattractant protein 3 (MCP-3) (Choi et al., 2001).

This result may be explained by the fact that periodontal pathogens, such as *Porphyromonas gingivalis*, participate in the activation of B lymphocytes and plasma cells to secrete specific IgG, IgA, and IgM antibodies that are involved in periodontitis pathogenesis (Seymour et al., 1983; Zouali, 2017). In addition, it is known that plasma cells produce a variety of pro-osteoclastic factors, such as IL-6, IL-10, Transforming Growth Factor alpha (TGF-α), TGF-β, and matrix metalloproteinases (MMPs), which facilitate the degradation of periodontitis tissues. In turn, a variety of cytokines are required for the growth and differentiation of B cells, including receptor activator of NF-kappa B ligand (RANKL), B cell activating factor (BAFF)and tumor necrosis factor ligand superfamily member 13-like (Zouali, 2002, 2017). During subgingival biofilm stimulation, neutrophils are upregulated and produce various proinflammatory mediators, reactive oxygen/nitrogen species (RONS), and metalloproteases (MMPs) to degrade the extracellular matrix and enhance osteoclastic activity (Sima et al., 2019).

Furthermore, our data identified that the T/B cell ratio was downregulated in periodontitis-affected tissues, which suggested a dysregulation of immunity homeostasis. Regulatory T cells, which played a protective role in periodontitis, were similarly impaired in rats with periodontitis during pregnancy, which exacerbated the bone loss in periodontitis (Hays et al., 2019). Consistent with our study results, CD8 + T cells, which directly inhibit osteoclast formation, were found to be inhibited in periodontitis lesions (Choi et al., 2001).

Recent studies have revealed that osteoclasts in periodontitis tissues gave rise to this phenomenon. In a chronic inflammation state, osteoclasts in periodontal bone microenvironments contributed to the formation of an immunosuppressive microenvironment. This microenvironment inhibited the proliferation and polarization of naive T cells with dendritic cells by herpes virus entry mediator (HVEM), programmed death ligand 1 (PD-L1) and indoleamine 2,3-dioxygenase (IDO). In addition, osteoclasts, also function as antigen presenting cells (APCs) and induced TNF-α production in T cells and helper T cells to moderate inflammation in periodontitis lesion (Madel et al., 2019).

### Differences in Immune Genes Expression Inhibited the Immune Response in Periodontitis

Differently expressed immune-related genes in gingival tissues with periodontitis versus healthy tissues altered the immune signaling pathway. A negative correlation among enriched immune-related pathways in periodontitis revealed depressed immune activation. Not only T cells and B cells but also dendritic cells called antigen-presenting cells were required for antigen-specific immune responses. Downregulated immune cell subpopulations inhibited the immune response in periodontitis.

Interestingly, a negative correlation was identified between periodontitis and the NGF pathway. The NGF pathway was involved in the repair and protection of neurons, which might function as a therapeutic target for Alzheimer’s disease (Zilony-Hanin et al., 2019). It has been shown that proinflammatory cytokines in periodontitis suppress the TGF-β-mediated expression of NGF by downregulating TGF-β-induced Smad2/3 and p38 MAPK signaling (Ohta et al., 2018). With emerging evidence indicating a relevant relationship between Alzheimer’s disease and periodontitis, the NGF pathway might play a role as a bridge in Alzheimer’s disease patients with periodontitis (Holmer et al., 2018; Dominy et al., 2019; Hayashi et al., 2019).

In addition, a close interrelation between periodontitis and diabetes was verified by a number of studies (Preshaw et al., 2012; Nascimento et al., 2018). Periodontal pathogenic substances such as LPS promote the development of insulin resistance (IR) through toll like receptor-4 (TLR-4) signaling in the liver (Ilievski et al., 2015). Consistently, our data further emphasized the inhibition of the insulin signaling pathway in periodontitis.

### Immune-Related lncRNAs Regulate the Immune Response in Periodontitis

Our bioinformatic analysis indicated that lncRNAs play an important role in the immune response in periodontitis. Network analysis results revealed that FAM30A was involved in the immune response in periodontitis, with B cell activation and immune-related gene alteration. In accordance with the present results, previous studies have demonstrated that FAM30A was highly expressed in B cells and participates in vaccine-elicited responses (de Lima et al., 2019). Immunoglobulin genes (IGHD, IGHM, IGHG2a, and IGHV) were co-expressed with FAM30A and located in the genomic vicinity in response to vaccination, which suggested a *cis*-regulatory function of FAM30A (de Lima et al., 2019). Furthermore, we first described the potential function of the lncRNAs (GUSBP11 and LINC00525) in the immune response. GUSBP11 and LINC00525 were found to participate in the development of several cancers. GUSBP11 overexpression accelerated the viability and invasion of gastric cancer cells (Zheng et al., 2019). Moreover, differentially expressed GUSBP11 was identified in head and neck squamous cell carcinoma (HNSCC) (Zheng et al., 2019). LINC00525 enhanced the stemness and chemoresistance of colorectal cancer through the miR-507/ELK3 axis (Wang et al., 2019).

Several other lncRNAs were found to promote the occurrence of periodontitis through different molecular mechanisms. A lncRNA antisense non-coding RNA in the INK4 locus (ANRIL), the first reported genetic risk factor for aggressive periodontitis, mediated the inflammatory response through the signal transducer and activator of transcription 1-alpha/beta (STAT1) signaling pathway (Schaefer, 2018). Additionally, lncRNA-POIR regulated osteogenic differentiation in the human periodontal mesenchymal stem cell inflammatory microenvironment (Wang et al., 2016).

Despite these promising findings, there were two limitations remain. Firstly, only one gene sample series (GSE16134) was identified in this study. More samples regarding gingival tissue with periodontitis are required. Secondly, coregulation between immune-related lncRNAs and genes lack definitive evidence. Further research should be undertaken to investigate functional validation experiments for immune-related lncRNAs in periodontitis.

## Conclusion

In summary, this study focused on transcriptome analysis of the immune response in periodontitis, and identified differentially expressed immune-related genes and their potential functions in periodontitis lesions. Furthermore, we suggest an active involvement of lncRNAs in the immune response in periodontitis and it might help to explain the regulation of the immune mechanism in the development of periodontitis.

## Data Availability Statement

The periodontitis-related microarray data set GSE16134 was downloaded from the Gene Expression Omnibus database (https://www.ncbi.nlm.nih.gov/gds/).

## Ethics Statement

The studies involving human participants were reviewed and approved by Shenzhen Baoan Women’s and Children’s Hospital. The patients/participants provided their written informed consent to participate in this study.

## Author Contributions

DW, PZ, and XZ designed this study. ZL and PZ retrieved the data. DL and FC analyzed the data. DW drafted the manuscript.

## Conflict of Interest

The authors declare that the research was conducted in the absence of any commercial or financial relationships that could be construed as a potential conflict of interest.

## Footnotes

1 http://cibersort.stanford.edu/

2 https://www.immport.org

3 https://ucscgenomics.soe.ucsc.edu/gencode/

## Supplementary Material

FIGURE S1 Flow chart of the study design.

FIGURE S2 PCA analysis in DEMGs.

TABLE S1 Immune related lncRNA between periodontitis and health.

TABLE S2 Gene signature matrix in 22 human immune cell subpopulations.
