# Supplementary figures and images for: Expression Profiling and Cell Type Classification Analysis in Periodontitis Reveal Dysregulation of Multiple lncRNAs in Plasma Cells
Source: Front Genet. 2020 Apr 28;11:382. doi: 10.3389/fgene.2020.00382 (PMC7199422; doi:10.3389/fgene.2020.00382)

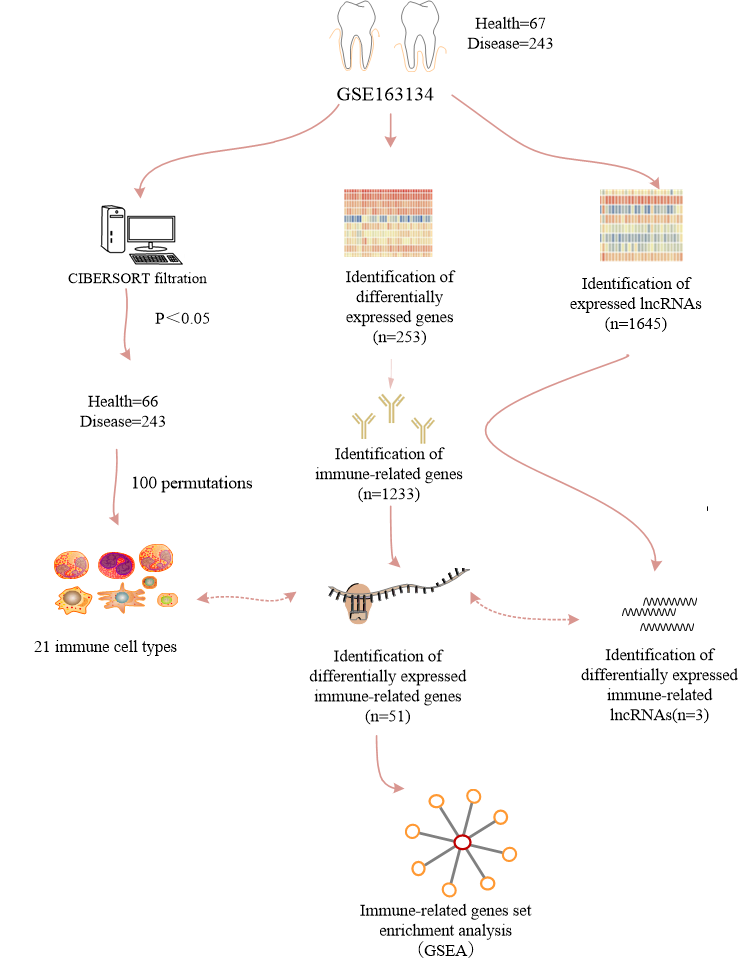

Supplement: FIGURE S1 — Flow chart of the study design. [file Image_1.TIF]

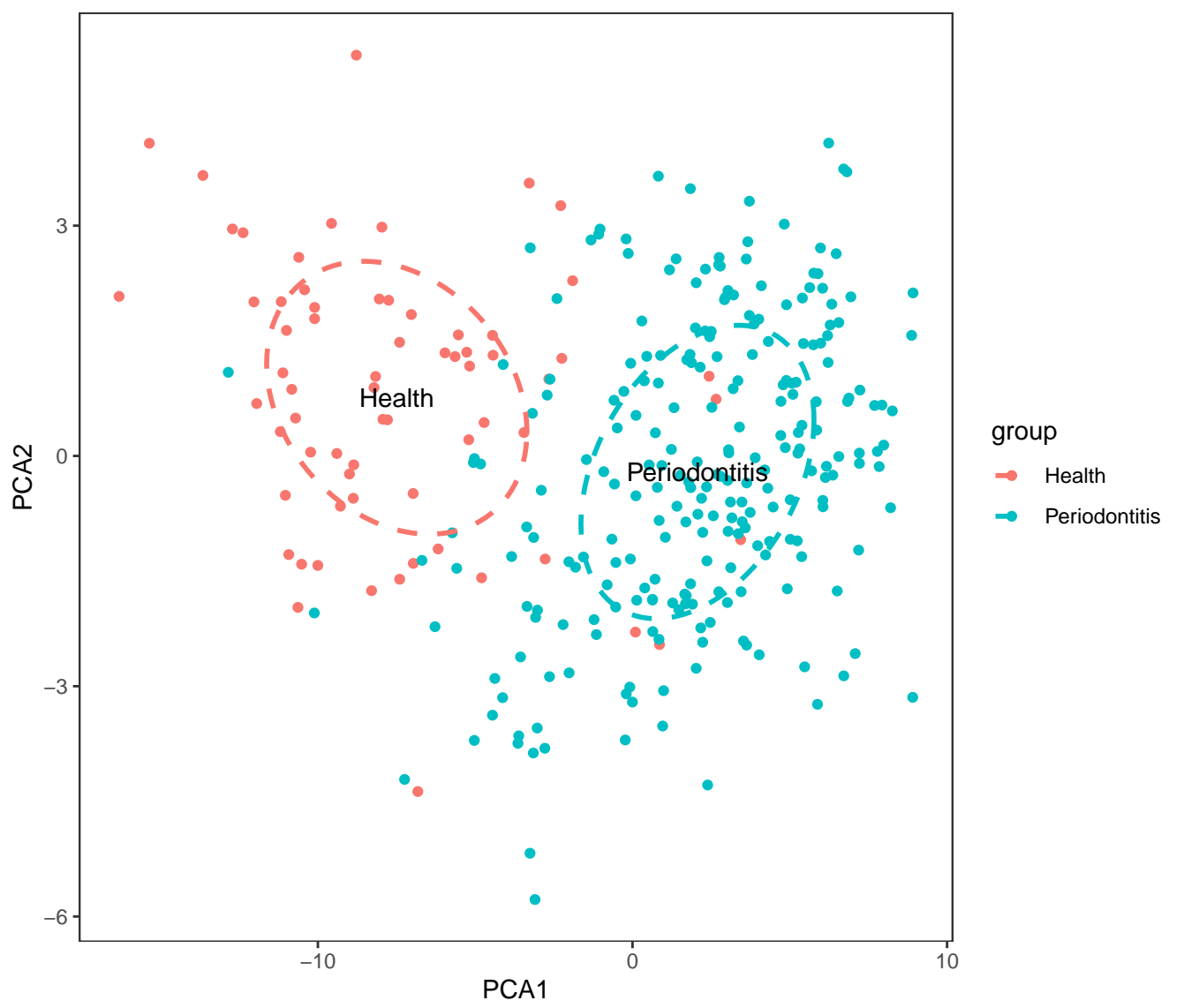

Supplement: FIGURE S2 — PCA analysis in DEMGs. [file Image_2.PDF]
