# Supplementary material for: Expression Profiling and Cell Type Classification Analysis in Periodontitis Reveal Dysregulation of Multiple lncRNAs in Plasma Cells
Source: Front Genet. 2020 Apr 28;11:382. doi: 10.3389/fgene.2020.00382 (PMC7199422; doi:10.3389/fgene.2020.00382)
Supplement: TABLE S1 — Immune related lncRNA between periodontitis and health. [file Table_1.DOCX]

| **lncRNA** | **DEMGs** | **P value** | **R** |
| --- | --- | --- | --- |
| FAM30A | TNFRSF17 | 4.00E-97 | 0.915243122 |
| FAM30A | HLA-DOB | 1.16E-88 | 0.899556138 |
| FAM30A | PLCG2 | 2.39E-85 | 0.892603895 |
| FAM30A | PNOC | 7.70E-85 | 0.891495451 |
| FAM30A | PDK1 | 7.75E-76 | 0.869648076 |
| GUSBP11 | IGLV1-44 | 2.82E-74 | 0.86540278 |
| GUSBP11 | IGKC | 5.36E-73 | 0.861810043 |
| FAM30A | FCGR2B | 7.00E-72 | 0.858592071 |
| GUSBP11 | IGLJ3 | 9.74E-68 | 0.845906659 |
| GUSBP11 | IGLC1 | 2.99E-66 | 0.841053166 |
| GUSBP11 | IGHM | 1.08E-59 | 0.81754421 |
| FAM30A | IL16 | 3.52E-57 | 0.807531685 |
| FAM30A | IL10RA | 3.86E-57 | 0.807364843 |
| GUSBP11 | IGHD | 8.53E-54 | 0.793045496 |
| FAM30A | BMP6 | 1.09E-53 | 0.792572398 |
| FAM30A | CD19 | 1.46E-51 | 0.78280329 |
| FAM30A | CD79A | 4.51E-49 | 0.770690487 |
| FAM30A | PIK3CG | 1.02E-47 | 0.763769659 |
| GUSBP11 | IGHG1 | 2.77E-46 | 0.756178854 |
| FAM30A | IGLV1-44 | 3.70E-46 | 0.755504873 |
| GUSBP11 | ICAM2 | 7.67E-46 | 0.753781797 |
| GUSBP11 | CD79A | 8.59E-46 | 0.753514184 |
| FAM30A | IGLJ3 | 1.47E-45 | 0.752230206 |
| FAM30A | RAC2 | 7.79E-43 | 0.736709321 |
| FAM30A | IGHD | 1.62E-40 | 0.722564406 |
| GUSBP11 | HLA-DOB | 4.41E-39 | 0.713352093 |
| GUSBP11 | IL16 | 7.69E-38 | 0.705074891 |
| FAM30A | ICAM2 | 1.16E-37 | 0.703860529 |
| LINC00525 | FCGR2B | 7.94E-37 | 0.698092637 |
| LINC00525 | PDK1 | 1.10E-36 | 0.697102899 |
| FAM30A | CSF2RB | 2.19E-35 | 0.68781592 |
| LINC00525 | PNOC | 2.31E-35 | 0.687645917 |
| GUSBP11 | RAC2 | 4.55E-35 | 0.685489818 |
| FAM30A | IGHM | 6.89E-35 | 0.684161803 |
| GUSBP11 | IL2RG | 1.91E-34 | 0.680867848 |
| LINC00525 | PIK3CG | 4.34E-34 | 0.678179703 |
| GUSBP11 | CD19 | 1.14E-33 | 0.674987378 |
| FAM30A | IGHG1 | 1.01E-32 | 0.667624769 |
| LINC00525 | PLCG2 | 4.65E-32 | 0.662328759 |
| GUSBP11 | ANXA6 | 1.41E-31 | 0.658405412 |
| LINC00525 | HLA-DOB | 7.13E-31 | 0.652595174 |
| GUSBP11 | TNFRSF17 | 8.99E-31 | 0.651754358 |
| GUSBP11 | IL10RA | 2.00E-29 | 0.640208127 |
| LINC00525 | TNFRSF17 | 2.41E-29 | 0.639498566 |
| GUSBP11 | PLCG2 | 3.90E-29 | 0.637662288 |
| GUSBP11 | PNOC | 1.46E-28 | 0.632551466 |
| GUSBP11 | C3 | 2.72E-28 | 0.630091436 |
| FAM30A | CXCR4 | 1.10E-25 | 0.605341734 |
| GUSBP11 | CD14 | 1.21E-25 | 0.604927632 |
| FAM30A | ANXA6 | 3.29E-25 | 0.600568687 |
| FAM30A | NPR3 | 9.26E-27 | -0.615813429 |
| GUSBP11 | RORA | 2.41E-28 | -0.630581942 |
